# Radiocarbon and wood anatomy as complementary tools for generating tree-ring records in Bolivia

**DOI:** 10.3389/fpls.2023.1135480

**Published:** 2023-02-23

**Authors:** Arturo Pacheco-Solana, Rose Oelkers, Rosanne D’Arrigo, Guaciara M. Santos, Milagros Rodriguez-Caton, Ernesto Tejedor, Eugenia Ferrero, Alfredo F. Fuentes, Carla Maldonado, Laia Andreu-Hayles

**Affiliations:** ^1^ Tree Ring Laboratory at LDEO, Columbia University, New York City, NY, United States; ^2^ Earth System Science Department, University of California, Irvine, Irvine, CA, United States; ^3^ Department of Plant Sciences, University of California, Davis, Davis, CA, United States; ^4^ National Museum of Natural Sciences-Spanish Research Council, Madrid, Spain; ^5^ Instituto Argentino de Nivología, Glaciología y Ciencias Ambientales, CONICET-Universidad Nacional de Cuyo, Mendoza, Argentina; ^6^ Herbario Nacional de Bolivia, Instituto de Ecología, Carrera de Biología, Universidad Mayor de San Andrés, La Paz, Bolivia; ^7^ Center for Conservation and Sustainable Development, Missouri Botanical Garden, St. Louis, MO, United States; ^8^ Centro de Investigacion Ecologica y Aplicaciones Forestales (Catalan Institution for Research and Advanced Studies), Barcelona, Spain; ^9^ Catalan Institution for Research and Advanced Studies (Catalan Institution for Research and Advanced Studies), Barcelona, Spain

**Keywords:** *Neltuma alba*, algarrobo blanco, bomb-pulse radiocarbon dating, tropical dendrochronology, Bolivia, South America

## Abstract

The science of tropical dendrochronology is now emerging in regions where tree-ring dating had previously not been considered possible. Here, we combine wood anatomical microsectioning techniques and radiocarbon analysis to produce the first tree-ring chronology with verified annual periodicity for a new dendrochronological species, *Neltuma alba* (commonly known as “algarrobo blanco”) in the tropical Andes of Bolivia. First, we generated a preliminary chronology composed of six trees using traditional dendrochronological methods (i.e., cross-dating). We then measured the ^14^C content on nine selected tree rings from two samples and compared them with the Southern Hemisphere (SH) atmospheric ^14^C curves, covering the period of the bomb ^14^C peak. We find consistent offsets of 5 and 12 years, respectively, in the calendar dates initially assigned, indicating that several tree rings were missing in the sequence. In order to identify the tree-ring boundaries of the unidentified rings we investigated further by analyzing stem wood microsections to examine anatomical characteristics. These anatomical microsections revealed the presence of very narrow terminal parenchyma defining several tree-ring boundaries within the sapwood, which was not visible in sanded samples under a stereomicroscope. Such newly identified tree rings were consistent with the offsets shown by the radiocarbon analysis and allowed us to correct the calendar dates of the initial chronology. Additional radiocarbon measurements over a new batch of rings of the corrected dated samples resulted in a perfect match between the dendrochronological calendar years and the ^14^C dating, which is based on good agreement between the tree-ring ^14^C content and the SH ^14^C curves. Correlations with prior season precipitation and temperature reveal a strong legacy effect of climate conditions prior to the current *Neltuma alba* growing season. Overall, our study highlights much potential to complement traditional dendrochronology in tree species with challenging tree-ring boundaries with wood anatomical methods and ^14^C analyses. Taken together, these approaches confirm that *Neltuma alba* can be accurately dated and thereby used in climatic and ecological studies in tropical and subtropical South America.

## 1 Introduction

As at higher latitudes, some tropical trees can experience cambial dormancy due to an array of adverse environmental conditions, although the annual growth boundaries may not be as clearly defined (Worbes, 2002). Seasonal river floodings and water salinity in mangrove forests (Worbes and Junk, 1989; Schöngart et al., 2002; Chowdhury et al., 2008), for example, can result in annual tree-ring formation in tropical species. An added complexity is that intra-seasonal fluctuations in climate can generate false rings, rings that wedge out, and/or other poorly defined anatomical ring boundaries (Priya and Baht, 1998; Worbes, 2002; Dünisch et al., 2003; Wils et al., 2009), challenging the identification and precise dating of annual growth layers. However, in most cases across the tropics, dry-season conditions are the main factor controlling annual tree growth (Zuidema et al., 2022). Annual formation of tropical tree rings has now been confirmed by radiocarbon dating for several tree species in tropical South America (Worbes, 2002; Dezzeo et al., 2003; Andreu-Hayles et al., 2015; Brienen et al., 2016; Santos et al., 2020, 2021; Rodríguez-Morata et al., 2022), revealing great unrealized potential for addressing important ecological and climatological questions across this vast region.

Precise calendar dating of tropical trees can be confirmed using radiocarbon analyses. Radiocarbon (^14^C) bomb pulse dating uses the so-called *^14^C bomb-peak*, an anthropogenically-derived spike in atmospheric concentration of ^14^CO_2_, to directly verify the calendar years determined by traditional dendrochronological techniques using the ^14^C signatures fixed in organic tissues, such as tree rings, after 1950 (Worbes and Junk, 1989; Reimer et al., 2004). The *^14^C bomb-peak* was formed by the rapid increase of atmospheric ^14^CO_2_ due to nuclear weapons testing in the 1950s and their ban in 1963. Ever since, the excess of atmospheric ^14^C has decreased due to oceanic and terrestrial uptake, fossil-fuel dilution, and atmospheric mixing. These abrupt shifts in ^14^CO_2_ concentrations can thus be used to independently validate tree-ring dating within one year or less of uncertainty (Santos et al., 2020). This type of independent validation has been successfully used to confirm the annual periodicity of calendar dates established using dendrochronological methods, in particular for tropical species (Biondi and Fessenden, 1999; Hua et al., 1999; Fichtler et al., 2003; Menezes et al., 2003; Westbrook et al., 2006; Biondi et al., 2007; Wils et al., 2009; Soliz-Gamboa et al., 2011; Andreu-Hayles et al., 2015; Santos et al., 2020, 2021; Ancapichún et al., 2021; Santos et al., 2022). The basic principle is that the process of photosynthesis fixes atmospheric ^14^CO_2_ in the cellulose of plants. The ^14^C signature measured in cellulose of individual tree rings thus serves as a reliable proxy of atmospheric ^14^CO_2_ at a given moment in time.

Another important method for facilitating the dating of tropical trees involves the investigation of anatomical characteristics of the wood. Such features can vary from macroscopical traits to very small and delicate details only observable under light transmission microscopes (Baas et al., 2004; Crivellaro and Schweingruber, 2015). The quality and the time needed to obtain permanently-fixed histological cuts from transverse sections of the stem has greatly improved with higher precision microtomes and image capturing instruments with higher resolution that allow for very precise anatomical measurements (Gärtner and Schweingruber, 2013). These technological advances in cutting and image capturing provide a much-improved analysis of subtle details of key tropical hardwood anatomical traits, which are crucial in defining tropical tree-ring boundaries.

The genus *Prosopis* has been recently disintegrated (Hughes et al., 2022) based on robust evidence which reported that it is polyphyletic, with three separate lineages including *Anonychium, Strombocarpa* and *Neltuma*. Therefore, our target species formerly known as *Prosopis alba* is now named as *Neltuma alba* and we will use this name hereafter. When citing prior studies, the genus name *Prosopis* will still be used to refer to other species as cited in the literature. In the former genus *Prosopis*, the identification of annual growth rings has historically been challenging. This is partly due to the wide diversity in growth patterns among the species within this genus, but also due to the anatomical variability within the same species or even within the same specimen (Villalba et al., 2000). Nevertheless, there has been success in building tree-ring width chronologies for dendroclimatology using different species of this genus in hydrological (*Prosopis pallida* and *Prosopis ferox)*, ecological (*Prosopis tamarugo* and *Prosopis flexuosa)* and anatomical studies (*Prosopis caldenia* and *Prosopis ferox)* (Giantomasi et al., 2009; Ambite et al., 2022). Published studies of our target species *Neltuma alba* are limited to only one mention of a floating chronology cited as *Prosopis alba* in (Rivera, R. and Shea (2010). This 90-year record was produced from archeological material from Chile and was dated to an uncalibrated ^14^C age of 2530 ± 40 BP obtained using Accelerator Mass Spectrometry or AMS.

**Figure 1:**
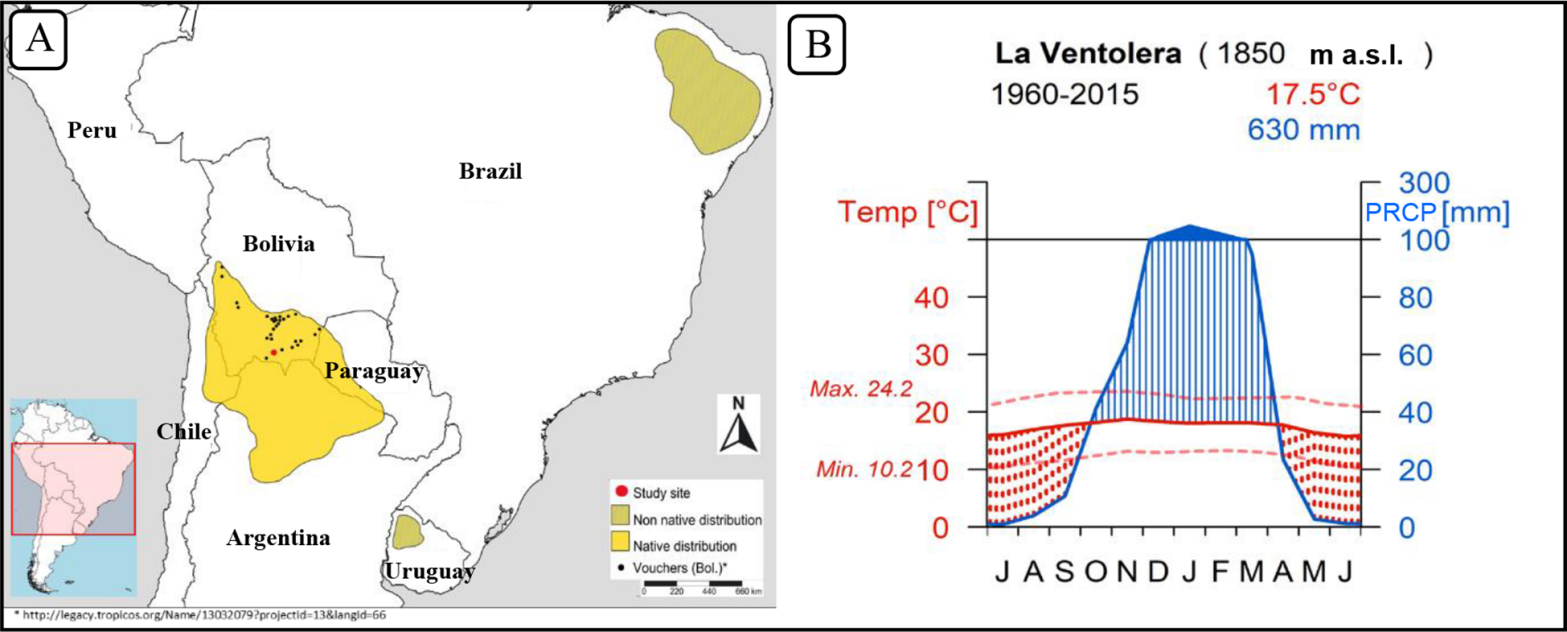
*Neltuma alba* species distribution map **(A)** and climograph **(B)** of the study site at La Ventolera, Bolivia.

*Neltuma alba* is widely distributed across arid and semi-arid tropical and sub-tropical South America. Its native distribution spans along the semi-dry inter-Andean valleys in Bolivia, Chile and Argentina, and expands into the Chaco regions of Argentina and Paraguay (**Figure 1**). It is considered a species of major economic significance as it provides timber, edible fruits, forage, and ecosystem services (Fontana, 2020). Within its distribution, *Neltuma alba* [cited as *Prosopis alba* in Verga et al. (2009)] features several morphological subspecies. The chronology described herein is the first of its kind for this species and it is located at the northernmost part of its natural range (**Figure 1A**). This forest population grows in the dry highlands of southern Bolivia, where there are well-defined dry and wet seasons (**Figure 1B**). These environments are known as Bolivian-Tucuman sub-andean and inter-andean phreatophytic (deep-rooted plants that obtains a significant portion of the water that it needs from the phreatic zone or zone of soil water saturation) forests (Navarro, 2011). There are formerly named *Prosopis* species that grow in Bolivia such as *Prosopis ferox* (Morales et al., 2001), which grows in prepunic (phytogeographic region characterized by xerophytic shrubs) altitudinal floors of Bolivia and Argentina forming floristic combinations with *Neltuma alba* (cited as *Prosopis alba* in Navarro (2011), and *Prosopis flexuosa* with annual periodicity demonstrated by traditional crossdating, mainly located at more xeric latitudes in the Argentinian Chaco region (Giantomasi et al., 2009).

The main objective of this study is to use radiocarbon analysis and wood anatomical techniques to assess the dendrochronological potential of previously uninvestigated tropical tree species like *Neltuma alba*. Specifically, our aims are (1) to produce the first tree-ring width chronology for *Neltuma alba* in Bolivia using complementary techniques such as radiocarbon and wood anatomy analyses, (2) to assess how *Neltuma alba* growth is influenced by climate variability. Our findings describe the climate sensitivity of *Neltuma alba*, its vulnerability to projected warming scenarios, and its future potential as a reliable proxy for climate reconstruction in the Bolivian and greater South American tropics.

## 2 Materials and methods

### 2.1 Target species, study area, and climate

Our target species is *Neltuma alba* (Griseb.) C.E. Hughes & G.P. Lewis (syn. *Prosopis alba* Griseb.) (Hughes et al., 2022), which has a geographic range broadly covering the Andean semi-arid mid-altitude plateaus located around the international borders between Bolivia, Chile, and Argentina, and extending south into the Chaco plains of Paraguay and north-central Argentina (**Figure 1A**). Due to its many economic uses, *Neltuma alba* has been planted in areas outside its native distribution (**Figure 1A**).

Our sampling site is located near La Ventolera (21°42’S, 64°29’ W) in the Tarija Department in Bolivia at an altitude of 1850 m a.s.l., within the dry inter-andean forest type. The specific area where our trees were growing was at the bottom of a valley on very flat terrain, which is a preferred ecological setting for *Neltuma alba*. The total annual precipitation is 630 mm, distributed mainly from October to April, with a prolonged dry season from May to September. The annual mean temperature is 17.5°C, without large seasonal variations along the year (**Figure 1B** **).** Sample collection was performed opportunistically during a field campaign in July 2019 at a site where many old individuals were being cut down to open an area for agricultural purposes. We collected 10 complete cross sections, mostly from the trunks and some branches from older-looking individuals. Coring this specie is possible but extremely hard and due to its stem eccentricity, cross sections are preferred.

### 2.2 Sample preparation and dendrochronological measurements

Cross sections were air-dried at room temperature and shipped to the Tree Ring Laboratory of the Lamont-Doherty Earth Observatory of Columbia University (NY, USA). Samples were sanded with increasingly fine grit sandpaper (120 to 1200 grit), first using an orbital sander and finally by hand. The sanded surface was deep cleaned using a stream of compressed air to clear out sand dust from vessels. Surfaces were subsequently liberally covered with chalk powder to increase the contrast between anatomical features (Arx et al., 2016). After dry cleaning the excess chalk, the samples were ready for initial visual crossdating under a stereomicroscope. We assigned to each annual ring the calendar year corresponding to the beginning of radial growth (Schulman, 1956). Specifically, as the sampling was conducted in July 2019, the most recent ring closest to the bark was dated as year 2018 when the tree began growing.

The samples were very eccentric with poor circular uniformity that led to very variable thickness of growth layers along the circumference (**Figure S1**). To facilitate the visual identification of the rings, we physically marked the clearest tree rings along their circumference for use as “reference years”. Using an EPSON Expression 11000XL scanner, we scanned 3 to 6 radii on each cross-section at a resolution of 3200 dpi. We measured the rings over these images using the software Coorecorder 9.5 (Larsson, 2014), and using its complementary program Cdendro 9.5 we obtained the ring-width timeseries for several selected radii on each cross-section. The resulting raw ring-width individual timeseries were statistically analyzed using the COFECHA software (Holmes, 1983) to control the quality of our visual crossdating and facilitate the identification of possible false or absent rings.

To remove trends and/or growth pulses caused by age, competition or disturbance we calculated individual ring-width indices (TRWi) that were averaged to develop the mean chronology for this site, quantifying growth variability at inter-annual, decadal and longer time scales following standard dendrochronological proceedings (Cook and Kairiukstis, 1990). First, we removed the size/age trends commonly observed in tree-ring width timeseries by fitting a cubic smoothing spline function with 50% frequency cut-off of 30 years. We then computed the ratio between the observed and fitted values to obtain detrended series (Cook and Kairiukstis, 1990). Finally, we averaged the detrended series *via* a bi-weight robust mean (Cook, 1985) to build the mean tree-ring width chronology using the R package dplR (Bunn, 2008).

### 2.3 Wood anatomy

We selected two trees from our best crossdated individuals and extracted a rectangular section of wood of 1 cm width and 1 cm height covering the entire sapwood section for each sample. We next divided the aforementioned pieces into 3–5 cm long segments which were boiled for one hour to soften in a 1:3 solution of water and glycerin. Using a slide microtome (Gärtner and Nievergelt, 2010) we cut transverse histological wood sections (15–20 µm thick) for image analysis. The microsections were stained using a mix of safranin (1%) and astra blue (0.5%) solutions in order to dye the cell walls containing lignin (red) or only cellulose (blue). The sections were then fixed and permanently mounted onto glass microscope slides using a synthetic resin [EukittTM, Quick-hardening mounting medium (Sigma-Aldrich)]. Digital images were captured using an AmScope 12 MP Color CMOS Digital Eyepiece Microscope Camera installed in a light transmission microscope (Leitz, Laborlux 11, Type 020-435.028) using magnifications of 40X, 100X and 200X. We created panoramic photographs stitching together multiple overlapping images using PtGui Pro (v. 8.3.3) software. This process allowed us to compare the anatomical and sanded scanned images used previously for measuring ring-width side-by-side.

### 2.4 Radiocarbon analysis

In order to independently validate our conventionally-dated ring-width chronology, we utilized high precision ^14^C bomb pulse dating analysis. From our two best-crossdated samples, we selected five individual rings formed before and after the ^14^C atmospheric peak. Using this approach, it was possible to assess if the ^14^C content of the wood material dated by dendrochronological methods matches the ^14^C curve at both sides of the peak, confirming the number of years that should be between each tree ring measured. This approach allows for the identification of potential dating errors within the period from the onset of the ^14^C spike to the present. At present the post-AD 1950 atmospheric ^14^C has been divided into different inter-hemispheric zones: NH1, NH2 and NH3 for the Northern Hemisphere and SH1-2 and SH3 for the Southern hemisphere (Hua et al., 2012; Hua et al., 2013), based on proxy evidence from around the globe.

We first cut 3mm thick slices of wood longitudinally from the two selected radii. Under a stereomicroscope, we separated each ring using a scalpel and chopped them into small pieces ranging from ∼2.0–3.0 mm long to 0.5–1.0 mm wide (e.g. Rodriguez-Caton et al., 2021, as an example). Extraction of α-cellulose was performed following the protocols developed at LDEO for stable isotope analysis in tree-ring cellulose (Andreu-Hayles et al., 2019). Any remnants of acetic acid and CO_2_ were eliminated by performing a final 30-minute bath of 1N HCL at 70°C followed by several ultra-pure (Mili-Q) water rinses (Santos et al., 2020). Careful ring separation is crucial to obtain precise results on the ^14^C dating, while fine chopping is essential to facilitate the effectiveness of chemicals during cellulose extraction. Each single cellulose sample was homogenized using a Fisher Scientific FS20 ultrasonic bath, frozen for a minimum of a 24h period and freeze-dried for another 24h. The resulting dried cellulose samples were then stored in an electric desiccator before shipment to the Keck Carbon Cycle Accelerator Mass Spectrometer (KCCAMS) at the University of California where radiocarbon measurements were conducted on graphite targets (Santos and Xu, 2017; Santos et al., 2020) following established protocols (Reimer et al., 2004). The ^14^C results are being reported as F^14^C (Fraction modern carbon).

### 2.5 Climate and growth relationships

Tree growth-climate relationships were calculated using correlation coefficients between *Neltuma alba* detrended ring-width chronologies and high resolution gridded temperature data from the Climatic Research Unit (CRU), University of East Anglia and NCAS (Harris et al., 2020). For precipitation, we used a precipitation product generated using the *reddPrec* R package (Serrano-Notivoli et al., 2017) based on all available local weather stations precipitation records. Bootstrapped Pearson correlation coefficients were calculated for the period 1960-2015 for a total of 24 months from July before the previous growing season until June after the targeted growing season. The previous growing season months were included to account for potential lag effects of climate on growth (Fritts, 1976). We performed the analyses using the R package *Treeclim* (Zang and Biondi, 2015) which provides significant values *via* stationary bootstrapped confidence intervals (Biondi and Waikul, 2004). We also calculated moving correlations using periods of 35-year windows lagged 1-year at a time to assess the stability of the climate-growth relationships along the whole period. To address the potential covariance of temperature and precipitation, we applied the *Seascorr* function (Meko et al., 2011; Zang and Biondi, 2015). This function provides a method in which partial correlations remove the influence of the covariance between the two climate components when calculating their respective coefficients of correlation with tree-ring width. The following formulas show how this is achieved:


(1) $$r_{PCr.T}=\frac{r_{PCr}-r_{TP}r_{TCr}}{\left[\sqrt{(1}-r_{TP}^{2})\right]\left[\sqrt{(1}-r_{T.Cr}^{2})\right]}$$

(2) $$r_{TCr.P}=\frac{r_{TCr}-r_{TP}r_{PCr}}{\left[\sqrt{(1}-r_{TP}^{2})\right]\left[\sqrt{(1}-r_{P.Cr}^{2})\right]}$$

where *r_PCr.T_* and *r_TCr.P_* represent the partial correlations between the climate parameters, precipitation (P) and temperature (T), and the tree-ring chronology (Cr) eliminating the influence of its climatic counterpart. Pearsons correlation coefficients calculated between the tree-ring chronology (Cr) and the climatic variables (T and P, respectively) are *r_TCr_* and *r_PCr_*, respectively, while *r_TP_* is the Pearson correlation between T and P. It is noteworthy that the absolute values of partial correlations are by design lower than those from Pearson correlations. The significance of each (partial) correlation is evaluated using exact bootstrapping by circulant embedding of the tree-ring data (Percival and Constantine, 2006).

## 3 Results

### 3.1 Wood anatomy

**Figure 2:**
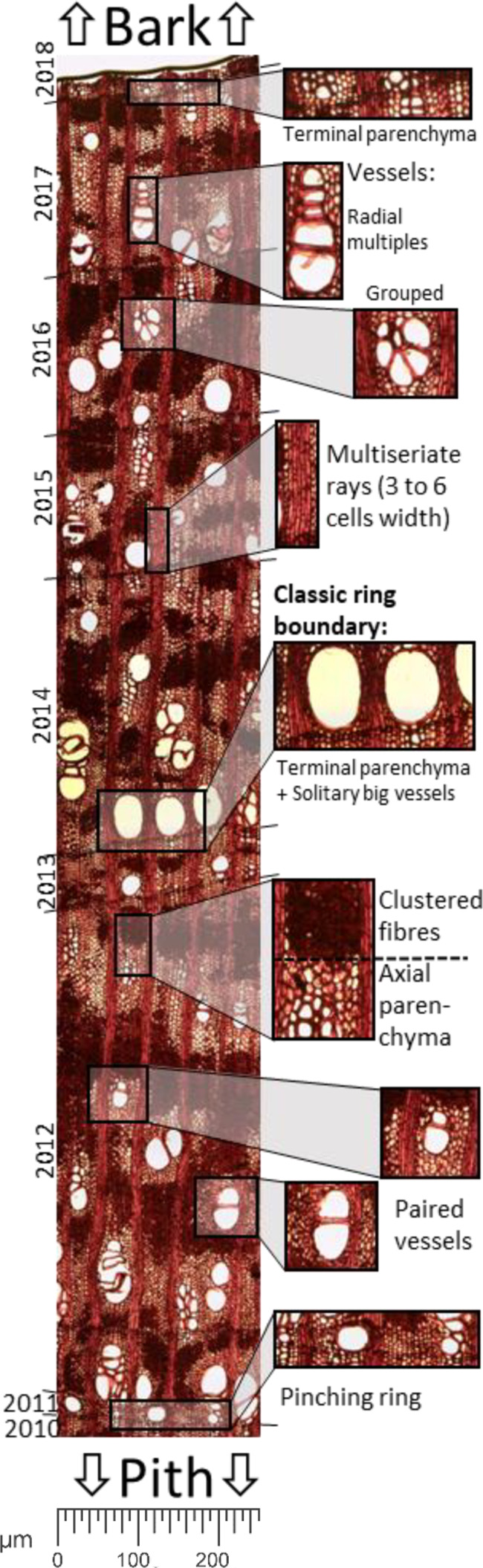
Panoramic image of a histological cut of a sapwood section of *Neltuma alba*. detailing the main wood anatomical traits used to take quantitative measurements and for differentiation of tree ring boundaries.

The wood of *Neltuma alba* is a semi-ring-porous arrangement of vessels, although in areas with narrow rings it approaches a diffuse-porous structure. Ring boundaries are generally defined by a clear line of terminal parenchyma cells of up to 3 layers and are generally followed by large solitary vessels in the contiguous earlywood. Paired, grouped and multiple radial vessels are common along the rest of the ring (**Figure 2**). The axial parenchyma (a tissue formed by thinner-walled elongate cells alive at maturity and associated with vessels) portrays a paratracheal vasicentric aliform parenchyma (wing shaped), disposed in wide and irregular bands. Together with dense clusters of fibers they form an irregular pattern, which fills most of the space of the larger rings. From a radial perspective the long multiseriate rays (3 to 6 cells wide) stretch out multidecadal lengths across the stem. In our samples the axial and radial parenchyma constitute around 40% of all xylematic tissue when analyzed in a transversal section.

**Figure 3:**
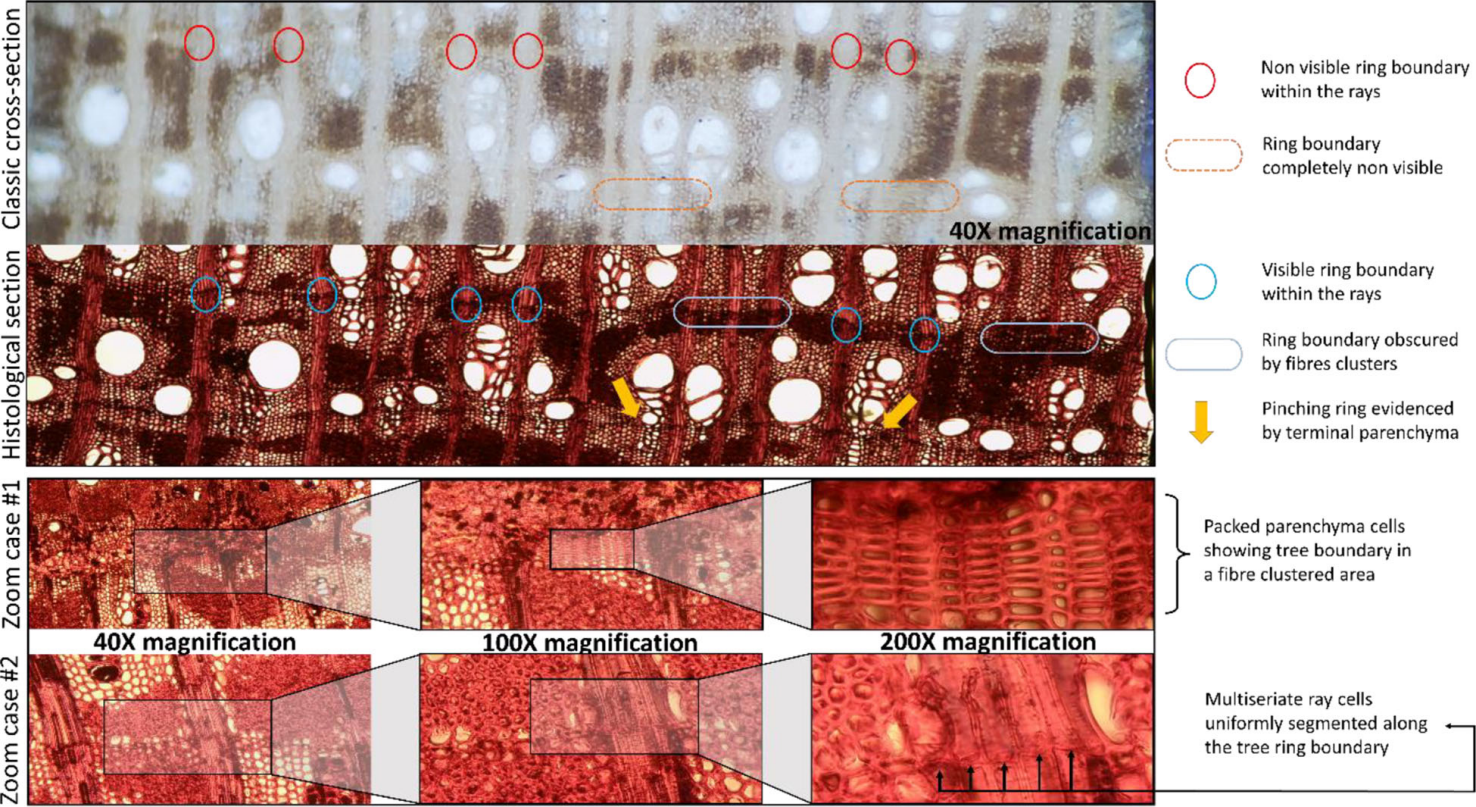
Comparison of distinctive diagnostic traits of ring boundaries as seen on the same section of a transversal cut of *N. alba* sapwood prepared with the classical approach of fine sanding viewed under a stereoscope and a histological cut viewed under a light transmission microscope. Below, two zoom cases of these anatomical traits are shown to high detail using a 200X magnification.

**Figure 3** shows a comparison between a classically prepared transverse section of sanded wood with chalk for dendrochronological analysis (above) and a histological section that has been dyed with safranin and astrablue and permanently fixed (below). Note that the key structures and traits that make identification of challenging tree-ring boundaries possible are only visible in the anatomical images. Very narrow and pinched-out rings (two or more rings which merge or separate at some point around the circumference of the stem), common in *Neltuma alba*, are impossible to detect *via* the classical approach, as the axial parenchyma blurs the thin line of terminal parenchyma (as seen in **Figure 3**). The absence of large solitary vessels in the narrow tree rings makes it even harder to identify these boundaries in a traditional approach in which the sample is only sanded and observations occurred under a stereomicroscope. In contrast, in histological sections (i.e., anatomically cut) terminal parenchyma is easily spotted by means of a light transmission microscope.

The use of the maximum magnification (200X) allows us to detect very subtle structures that delimit the tree-ring boundary, even when in the anatomical sections there are clusters of tightly-packed fiber cells that obstruct a clear visualization of tree-ring boundaries. **Figure 3** – zoom case #1 shows how in an area with particularly complex array of different types of cell clusters a very tight packed and axial parenchyma cells between such clusters of dark fibers can be observed. **Figure 3** - zoom case #2 shows how a uniform, segmented line across all cells of the multiseriate rays can be spotted where the tree-ring boundary is hidden within fiber clusters. These very specific details can only be observed thanks to the use of a light transmission microscope for visualization of the histological sections, which was critical for conducting a precise identification of tree-ring boundaries in the sapwood.

### 3.2 The tree-ring width chronologies

The *Neltuma alba* tree-ring width chronology was generated from 23 radii from six out of ten tree cross sections collected. The other four sections did not show similar growth patterns during the initial visual and statistical crossdating and were not considered for further analyses. The first version of our tree-ring width chronology spanned from 1883 to 2018 with a series intercorrelation of 0.325, and an average length of 78 years. This first version of the chronology was obtained using a dendrochronological approach in which the measurement of the width of the rings was performed using the software Coorecorder over scanned images of each radii on each sample.

**Figure 4:**
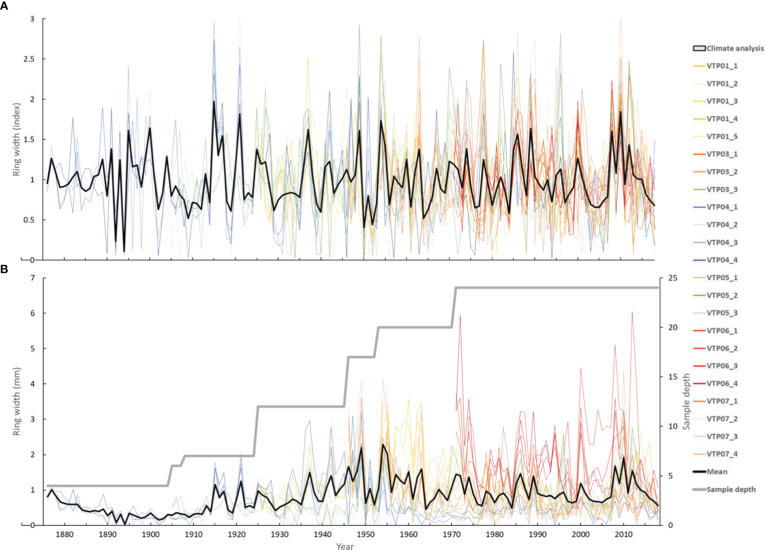
Ring width chronologies of 23 radius from 6 individuals of *Neltuma alba* shown as detrended index [above **(A)**] and raw measurements [below **(B)**] covering the period 1876-2018, with a common climate study period from 1960-2015.

An updated version of the chronology was generated after (i) determining the number of missing rings with ^14^C analyses (see section 3.3. below) and (ii) detecting the position of the missing rings using anatomical cuts visualized under a light transmission microscope for a better identification of subtle anatomical traits (see section 3.1 above). This updated version of the chronology spanned from 1876 to 2018 (**Figure 4**) and showed a series intercorrelation of 0.531, an average mean sensitivity of 0.541, an average length of 85 years, an Expressed Population Signal of 0.756 and a subsample signal strength (SSS) over 0.85 after 1944. These statistics indicated a relatively high common signal among trees. Note that the latter version of the chronology has a longer average length due to addition of missing rings on the sapwood section where most of the dating difficulties occurred (see section 3.3 below). Sapwood in *Neltuma alba* has a variable length in terms of number of rings as the border between this and the heartwood does not necessarily follow a single ring boundary but fluctuates all around its circumference (**Figure S1**). No missing rings were identified in the heartwood.

### 3.3 Radiocarbon (^14^C) analyses

**Figure 5:**
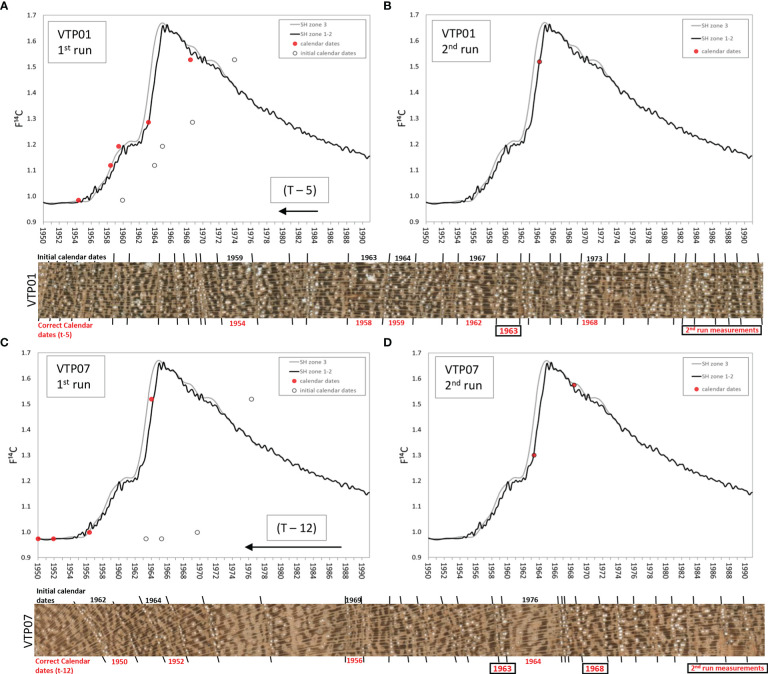
Radiocarbon test results of two sample trees [VTP01 **(A, B)** and VTP07**(C, D)**] before and after wood anatomy ring boundary analysis. Lines represent the calibration radiocarbon curve for the South Hemisphere while dots show single tree ring radiocarbon values (black circles show expected year values after classical crossdating and red dots represent actual calendar date values). Horizontal images show the original prepared wood material used for crossdating with the calendar dates assigned before the wood anatomy analysis (black) and after (red).

The radiocarbon analysis performed to validate the calendar dating of the first iteration of the ring-width chronology showed that the ^14^C content in the years 1959, 1963, 1964, 1967 and 1973 (Schulman years; **Table 1**) from the tree VTP01 were consistently offset by 5 years from the values of the SH ^14^C curves (**Figure 5A**). The tree-ring ^14^C values of samples from tree VTP07 had a consistent mismatch of 12 years from the SH ^14^C curve (**Figure 5C**) in the ring initially assigned to the calendar years 1962, 1964, 1969 and 1976 (Schulman years; **Table 1**). **Table 1** shows the calendar years ‘expected’ for the dendrochronological dates in order to obtain a good match between the tree-ring F^14^C values and the F^14^C SH curve zone 1-2 values.

**Table 1:** Radiocarbon analysis results for the two selected cross-sections of *Neltuma alba*.

TEST BATCH	TREE CODE	DENDROCHRONOLOGICAL CALENDAR DATES		RADIOCARBON DATA					CELLULOSE EXTRACTION DETAILS		
		(t) Year when tree start growing austral spring -Schulman convention -	(t+1) Year when tree stop growing austral summer- fall.	Radiocarbon tree-ring results			F^14^C curve values (Hua et al., 2022) SH Zone 1-2		Wood (mg)	Cellulose (mg)	Extraction yield (%)
				F^14^C	± 1σ	Expected year (t+1) ^14^C	Feb (t+1)	Average (GS) Oct (t) to Apr (t+1)			
1^ST^ RUN	VTP01	1959	1960	0.9846	0.0019	t-5 (1954)	1.1942	1.1917	88.4	36.25	41.01
	VTP01	1963	1964	1.1190	0.0020	t-5 (1958)	1.6157	1.5996	103.5	40.14	38.78
	VTP01	1964	1965	1.1927	0.0031	t-5 (1959)	1.6683	1.6681	87.9	32.74	37.25
	VTP01	1967	1968	1.2860	0.0027	t-5 (1962)	1.5841	1.5865	73.7	29.04	39.40
	VTP01	1973	1974	1.5282	0.0028	t-5 (1968)	1.4577	1.4592	41.6	14.08	33.84
	VTP07	1961	1962	0.9738	0.0018	t-12 (1950)	1.2123	1.2118	97.6	31.79	32.57
	VTP07	1963	1964	0.9736	0.0019	t-12 (1952)	1.6157	1.5996	58.3	21.36	36.64
	VTP07	1967	1968	0.9991	0.0019	t-12 (1956)	1.5841	1.5865	97	32.38	33.38
	VTP07	1975	1976	1.5200	0.0028	t-12 (1964)	1.4001	1.4018	73.8	22.62	30.64
2^ND^ RUN	VTP01	1963	1964	1.5185	0.0021	match	1.4859	1.4633	48.4	17.4	35.9
	VTP07	1962	1963	1.3004	0.0020	match	1.3928	1.3753	44.6	13.5	30.3
	VTP07	1967	1968	1.5750	0.0023	match	1.5725	1.5752	34.4	11.2	32.4
EXTRA RUN	VTP05	1962	1963	1.2925	0.0018	match	1.3928	1.3753	22.1	5.797	26.2

The 1^st^ and 2^nd^ run indicate if the measurements were done before and after the wood anatomy analysis, respectively. The two dendrochronological calendar dates columns show the growing season of *Neltuma alba*, starting approximately in October (t, Schulman year) and stopping around April (t+1, Schulman year). Based on Hua et al., 2022 dataset, we applied a monthly adjustment of 0.1250 to each calendar year (t+1) corresponding to the middle of the rainy season of our site (i.e., February 15^th^) to plot the tree-ring F^14^C over the SH ^14^C curves. The F^14^C values from the SH ^14^C curves Zone 1-2 are indicated for February 15th and for the average from October to April. Our F^14^C results include their standard deviation and the number of years offset in tree-ring F14C initially in relation to the SH ^14^C curves. Cellulose extraction details are also presented with the individual extraction yields. All post-bomb ^14^C results are shown as F^14^C signatures (Fraction Modern Carbon), as recommended in Reimer et al. (2004).

It was necessary to reexamine the wood material from these two trees through anatomical sections to visually detect and correctly identify the previously non-visible tree-ring boundaries (**Figure 3**). After the correct identification of the missing rings in the two samples with histological images, the rest of the samples of the chronologies were re-evaluated and the missing rings identified. All the rings were remeasured to develop new individual time series that were averaged and the final version of the tree-ring width chronology was created (**Figure 4A**). New tree-ring material was then sent for a second run of ^14^C analyses. The tree-ring ^14^C values, VTP01 (1963 = F^14^C: 1.5185; **Figure 5B**) and VTP07 (1962 and 1967 = F^14^C: 1.3004 and 1.5750, respectively, **Figure 5D**), matched relatively well with the ^14^C SH curve zone 1-2. This demonstrates that the calendar corrections after the identification of missing rings through wood anatomical techniques were accurate.

**Table 1** describes the ^14^C results. The dendrochronological dates were assigned to the year that the tree started growing based on the Schulman convention, while the ^14^C values use the calendar dates when the biomass was fixing ^14^CO_2_, often coinciding with the calendar year when the tree stopped growing. Therefore, both dates need to be well synchronized to avoid false mismatching when working in the South Hemisphere and tropical species that have growing seasons spanning across two calendar years, year t (i.e., when tree starts growing in austral spring) and year t+1 (when tree stops growing in austral summer-fall). Although no xylogenesis or phenological study has been made for this species in this area, we consider the growing season, when most wood material is produced, to approximately coincide with the rainy season that spans from October to April (**Figure 1B**). The cellulose extraction process showed that for the species *Neltuma alba* we can expect an extraction yield of 25 to 40% from the total wood weight (**Table 1**).

### 3.4 Tree-growth climate relationships

**Figure 6:**
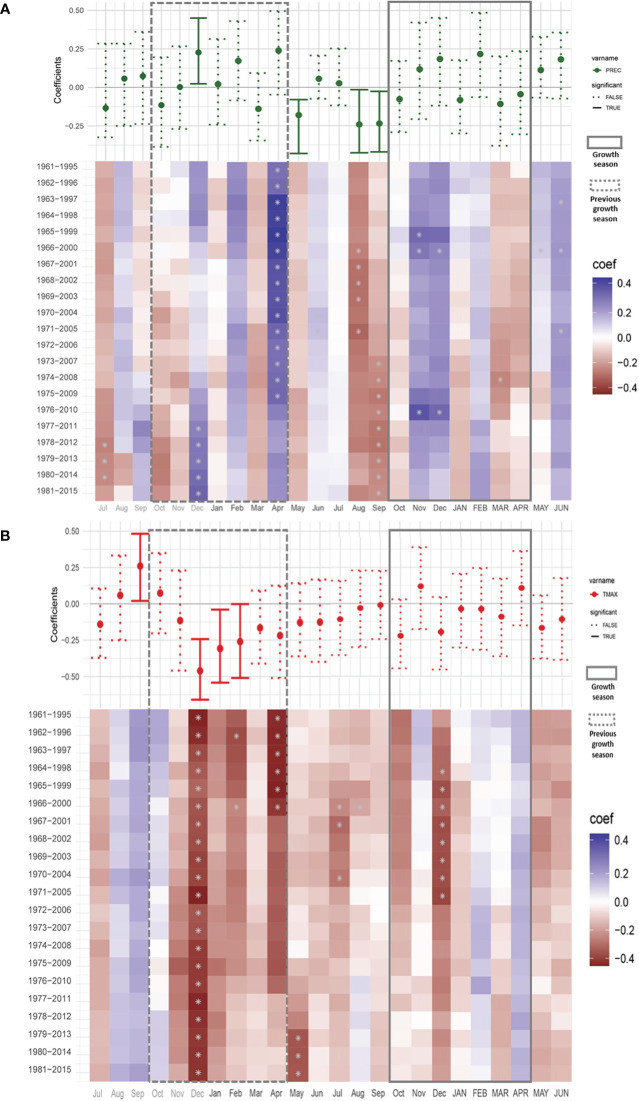
Climate–growth correlations between tree ring width and monthly climatic variables [Precipitation **(A)** and Tmax **(B)**] in *N. alba*. Correlations were calculated for a 24 months period starting in July before the onset of the previous growing season to June after the offset of the target growing season. Simple correlations are shown in a whisker format above a color matrix showing the dynamic correlations. Previous growing season is shown inside a discontinuous line rectangle (Oct – Apr) and the target growing season is surrounded by a continuous line rectangle (Oct – APR). Significance values where set at P< 0.05. Study period 1960-2015. *significant value.

We used the last version of the chronology (1876-2018) to perform a climate-growth analysis for the period from 1960 to 2015. This period was selected due to the availability of climate data for the region and the decrease of the sample size of the chronology in earlier periods. **Figure 6** shows the Pearson correlation coefficients between the *Neltuma alba* tree-ring width index (RWI) (**Figure 4A**) and monthly climate data (precipitation and maximum temperatures) spanning from the July prior to the previous growing season (grey colored) to June of the current growing season. During the previous growing season, December and April precipitation had a positive influence on growth (**Figure 6A**). Time-dependent correlations show that the significant correlations for April happened before 2010 and for December only during the 5 most recent intervals. In contrast, during the dry period before the growing season, particularly, August and September precipitation showed significant negative correlations with radial growth. There was no significant correlation with precipitation during the current growing season.

**Figure 7:**
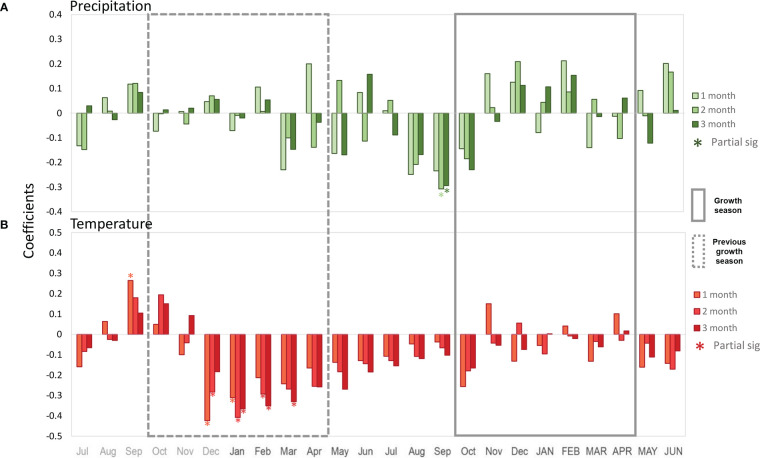
Covariance removed correlation coefficients between tree ring width index and two monthly climatic variables: precipitation (green **(A)**) and Tmax (red **(B)**); covering from July before the previous growing season to June after the target growing season. Bars indicate partial monthly correlations where the correlation of tree-ring width with precipitation and temperature was calculated after removing the covariability between temperature and precipitation, significant correlations are signed with an asterisk. Continuous and discontinuous grey rectangle lines indicate current and previous growing season respectively.

Comparison with maximum temperature showed a strong negative signal from December to February of the previous growing season (**Figure 6B**). The moving correlations showed stable and consistent significantly negative correlations for December, while for January and February the significance in the correlations was instable. Overall, the correlations between growth and temperature were negative from December of the previous year to December of the current year, albeit not always significant. **Figure 7** shows partial correlation coefficients for precipitation and maximum temperature (i.e., the correlations for each variable after removing its co-variability with the other for monthly, bi-monthly and three-monthly seasonal averages). For precipitation (**Figure 7A**), only August-September and July-August-September accumulated precipitation values prior to the growing season showed a negative partial significant (R=-0.31 p<0.05) correlation with *Neltuma alba* growth.

Regarding maximum temperature (**Figure 7B**), significant partial correlation coefficients were observed for December (R=-0.42 p<0.05) and January (R=-0.31 p<0.05) as shown with the simple correlation coefficients (**Figure 6**). Among the 2-month and 3-month seasonal averages, the highest partial correlations with *Neltuma alba* growth were observed with December-January (R=-0.40 p<0.05) and November to January maximum temperatures (R=-0.36 p<0.05). These results confirm the negative influence of maximum temperatures from the previous growing season on the growth of *Neltuma alba*.

**Figure 8:**
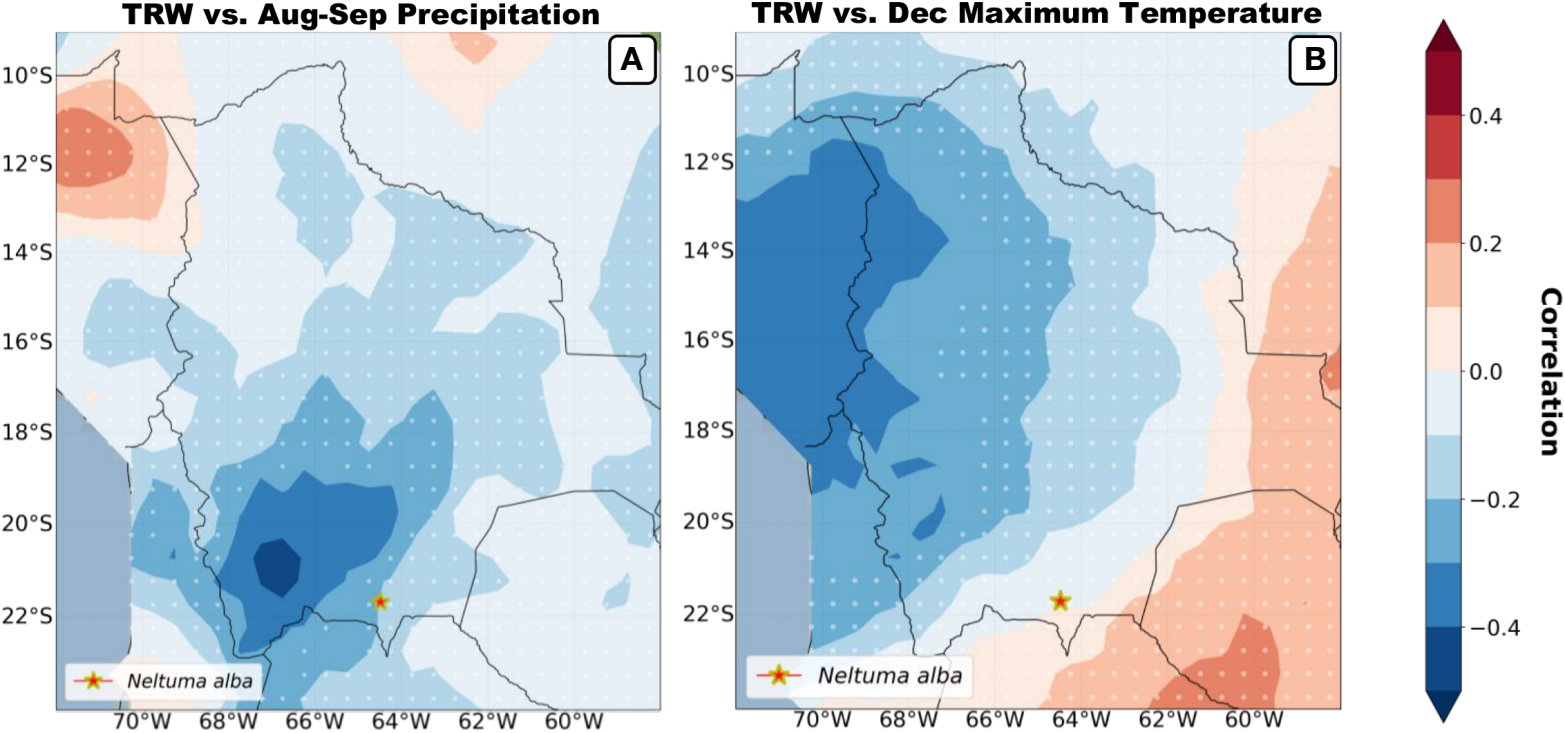
Spatial correlations (1960-2015) of mean tree ring width vs. precipitation and maximum temperatures is plotted based on the highest seasonal correlations shown in figure 7. Correlations were performed using CRU TS4.06 Precipitation during the period July to September **(A)** and CRU TS4.06 Tmax during the month of December **(B)** both covering the period previous to the growing season. The red star signals the sampling site of the studied species and the white dotted background fillings signal non-significant spatial correlations. *Location of our site.

Regional spatial correlations between the *Neltuma alba* tree-ring width chronology and CRU TS4.05 precipitation (**Figure 8A**) and CRU TS4.05 December maximum temperature (**Figure 8B**) for the period 1960-2015 were computed. The seasonal averages were chosen based on the highest correlations from **Figure 7**. *Neltuma alba* growth variability was significantly (p<0.05) negatively correlated with August-September precipitation close to our study site (**Figure 8A**) and with December maximum temperature on a larger scale over the Peruvian region (**Figure 8B**). Overall, significant negative correlations were obtained for both precipitation and temperature previous to the growing season in the Altiplano and central Andes regions adjacent to our study site.

## 4 Discussion

### 4.1 Combining dendrochronology, radiocarbon and wood anatomy analyses

This study illustrates the value of using multidisciplinary yet complementary approaches to investigate the dendrochronological potential of understudied tree species growing in complex environments like the tropics. In this case, the identification of annual growth rings in *Neltuma alba* trees in Bolivia was only possible when we combined: (1) classical dendrochronological methods based on the agreement in tree-growth variability among individual trees; (2) radiocarbon analysis of individual tree rings to independently validate calendar dates; (3) and finally, the detailed observation of anatomical features of the wood that allowed for accurately determining all the tree-ring boundaries of the samples.

Although standard tree-ring protocols recommend the use of two cores from each tree to account for internal variability within samples (Cook and Kairiukstis, 1990), this would not have been sufficient for detecting all the partial missing rings (i.e. absent) and false rings for a tree species like *Neltuma alba*. Due to the eccentricity of the radial growth in the stem and the presence of extremely narrow and wedging rings, working with cross-sections is essential for improved visualization of *Neltuma alba* wood (Worbes, 2002). Similar to Andreu-Hayles et al. (2015), we found that selecting three to five radii on each cross-section to perform visual cross-dating ensured that any absent or double rings could be identified by comparing with the other radii.

Radiocarbon analyses suggested calendar dates assigned in the first chronology using dendrochronological methods (i.e. visual crossdating) were offset by several years. As noted, our selected dendrochronologically dated tree-ring samples from the two individuals with the highest correlated radii resulted in consistent offsets of 5 and 12 years in each radius (**Figure 5**). The fact that the F^14^C values of the selected tree rings reproduce the shape of the bomb peak validates the annual periodicity of the *Neltuma alba* growth pattern. This finding was also crucial in revealing that the calendar dating errors were present only in the most recent years in both samples. The histological sections from the same measured radius confirmed that all the rings missed during the initial visual crossdating over the sanded samples occurred within the sapwood (**Figure 3**).

### 4.2 Sapwood visualization issues and anatomical cuts

As for other *Neltuma* species (i.e., previously named as genus *Prosopis* (Hughes et al., 2022)), the earlywood of most annual rings is characterized by the presence of large vessels that diminish in size towards the latewood as the amount of fiber tissue increases, followed by a thin band of terminal parenchyma tissue that ends the growth layer. Nevertheless, these general features described in literature (Gimenez et al., 1998; Bolzón de Muniz et al., 2016) were not easily visible in our samples. In particular, rings located in the sapwood portion of the sanded samples of *Neltuma alba* proved to be especially difficult. The part of the stem corresponding to the sapwood has a much lighter color than the heartwood, those prevalent light colors along the sapwood may complicate the identification of such anatomical traits.

Sapwood contains a large amount of starch and other components associated with the living processes of the trees’ transportation system, whereas the heartwood is a dead, mainly non-conducting tissue containing tannins and other substances that make it darker and harder, and represents the critical support structure of the tree (Taylor et al., 2002). The physical and chemical properties of these two tissues result in different type of reflective surfaces, which in the case of the sapwood leads to the inability to recognize some tree-ring boundaries under a simple microscope. The use of micrometric cuts over the sapwood allows us to clearly detect the full range of anatomical traits needed to correctly identify all the tree-ring boundaries.

The use of anatomical sections to better detect tree-ring boundaries was also especially useful when identifying the thin cells of terminal parenchyma blurred by dense fiber tissue. In particular, this was the case for very narrow rings that were not showing the expected line of large vessels at the start of the growing season (Villalba et al., 2000), but still formed a subtle but consistent line of terminal parenchyma. In some of the most challenging rings, we explored other diagnostic anatomical traits at an even smaller scale to facilitate the location of the ring boundary. As seen in **Figure 3** using a magnification of 100-200X, it is possible to see the coordinated segmentation of each cell of a multiseriate ray along the same position where the narrow band of terminal parenchyma cells is placed. This newly reported anatomical characteristic can be very useful when the amount of clustered fiber cells around the terminal parenchyma hides it from view. Overall, wood anatomical microsectioning was the only technique that allowed for the visualization and thus, correct identification, of all the tree-ring boundaries in the *Neltuma alba* samples analyzed here.

### 4.3 Climate influence on *Neltuma alba* radial growth patterns

Precipitation in our study region features a clearly defined annual seasonality that, in combination with subtle annual temperature variations, modulate radial growth of our target species *Neltuma alba*. Our findings indicate that Neltuma alba tree-ring width was negative correlated to maximum temperature variability from the previous-year three peak months of the rainy season, when maximum temperatures also exhibit the lowest values of the year (**Figure 6**). Similar results have been reported for *Prosopis flexuosa* growing in the Chaco and Monte regions of Cordoba and Mendoza, Argentina (Boninsegna and Villalba, 1989; Giantomasi et al., 2009), as well as for *Prosopis ferox* in Quebrada de Humahuaca, Argentina (Morales et al., 2004). These results indicate that lower maximum temperatures during the rainy season may allow the tree to avoid excessive evaporation, and/or may also permit a better replenishment of the water table in the bottom flat valleys where *Neltuma alba* prefers to grow. Other studies have shown how similar species in the former *Prosopis* genus have evolved to take advantage of water table levels, and in many cases their growth dynamic and colonization of new areas is driven by changes of underground water movement (Bogino and Jobbágy, 2011; Olson et al., 2020). *Neltuma alba* could be also driven by this environmental factor and this could very well explain the delay response in growth as previous season weather conditions will determine the availability of the water resource in the following growing season. This finding shows how even small intra-annual variations in climate can trigger significant responses in tree growth. Similar behavior could be present in other tropical species where intra-annual seasonality may not be as marked as in temperate zones.

Considering the specifics of the climate in our study area, we assess climate sensitivity to growth after removing the covariance between precipitation and temperature (**Figure 7**). The correlation patterns and significance of the partial correlations mirrored those from the simple correlation analysis ensuring the robustness of the results, which are not a mere consequence of an association between temperature and precipitation. This was not the case for precipitation during the peak of the previous rainy season (December) that correlated positively with tree-ring width (**Figure 6A**), but that lost any significance when partial correlations were calculated (**Figure 7A**). This suggests that the simple Pearson correlations with December precipitation was the result of the co-variance between precipitation and temperature, as opposed to a clear precipitation signal. Moreover, the results from single month to 3-month seasonal averages identified the most significant seasons, discarding any potential artifacts of the analyses. It is important to note that neither rainfall nor temperature showed significant correlations with growth during the current growing season. However, we did observe climate conditions that appeared to be particularly important prior to the growing season, namely significant negative correlations with precipitation (dry season).

An unexpected result of this study was the negative correlation of precipitation with growth just before the onset of the growing season. In other studies (López et al., 2005; López et al., 2006; Salazar et al., 2018; Nogueira et al., 2019) unseasonal rains resulted in wider rings. However, most of these studies were conducted along the coast of Peru where El Niño events led to heavy rainfall, while in contrast on the eastern side of the Andes near our study site, El Niño is associated with drought conditions. A hypothesis for *Neltuma alba* growth would take into account the significant positive correlation with temperature just before the previous growing season (**Figure 6B**) and how this could be related to a rapid and dramatic change in the prevalent conditions during the dormancy period that can act as a signal that may trigger growth onset. For similar species as *Prosopis flexuosa* an optimal thermal condition has been reported as an essential event to trigger the development of buds and reactivation of cambial growth (Giantomasi et al., 2009). If a similar process occurs in *Neltuma alba*, it could also explain the unexpected negative correlation with dry season precipitation before the growing season. This could be related to temperatures not reaching the tipping point for triggering growth onset because of general cloudy conditions. Therefore, the onset of growth would be delayed or hampered from a vigorous start.

Spatial correlations maps (**Figure 8**) show the broad regional strength recorded by the *Neltuma alba* tree-ring width chronology for the previous growing-seasons’ December temperature and the precipitation signal of August-September before the current growing season. The field correlations show that our trees seem to be mostly affected by the climatic variation above the drier biomes of the Altiplano and the Andes cordillera, while no significant values arise over the Amazonian and Chaco basin despite the fact that the highest significant correlations are not directly above our sample site. This spatial pattern can be potentially influenced by the critical nature of microclimatic conditions combined with the limited number of meteorological stations in the region and distance between them in a highly variable topography.

### 4.4 Conclusions

Our work illustrates the value of radiocarbon and wood anatomical analysis as key tools to generate tree-ring chronologies with annual resolution in species with not well-defined annual growth boundaries, in this case Neltuma alba. Wood anatomical analysis has proven to be an effective approach for uncovering the cellular scale details that can determine annual ring boundaries in species with challenging wood anatomical characteristics and growth patterns. At the same time radiocarbon analysis using the bomb-pulse dating method is a precise and independent methodology for confirming dendrochronological calendar dates, as well as tree-ring annual periodicity. Yet, this combined approach has still rarely been applied to trees in tropical environments, perhaps as it is labour intensive. Our study is the first to combine these methodologies in Bolivia to generate a tree-ring width chronology from the species Neltuma alba. Future studies of this nature will help provide novel valuable insights into both anatomical and growth responses to climate in the Andes.

## Data availability statement

The raw data supporting the conclusions of this article will be made available by the authors, without undue reservation.

## Author contributions

AP-S, LA-H conceived and designed the study. RO, MR-C, ET, MC, EF performed field sampling. ET contributed with elaboration of the climatic data. AP-S performed dendro and wood anatomy analyses and data interpretation. RO, AP-S, did cellulose extractions. GS performed the radiocarbon analysis. All authors contributed to the interpretation of the overall data. AP-S, LA-H wrote the main part of the manuscript. All authors contributed to the article and approved the submitted version. 

## Supplementary material

Supplementary Figure 1 Cross section of a sample of Neltuma alba, showing the eccentricity of the stem circumference. Zoomed area **(A, B)** show the variable length in terms of number of rings as the border between sapwood and heartwood doesn’t necessarily follow a single ring boundary. Zoomed area **(C)** show an extreme case of this same inconsistency of the border between wood types.
