# Supplementary figures and images for: Radiocarbon and wood anatomy as complementary tools for generating tree-ring records in Bolivia
Source: Front Plant Sci. 2023 Feb 23;14:1135480. doi: 10.3389/fpls.2023.1135480 (PMC9997647; doi:10.3389/fpls.2023.1135480)

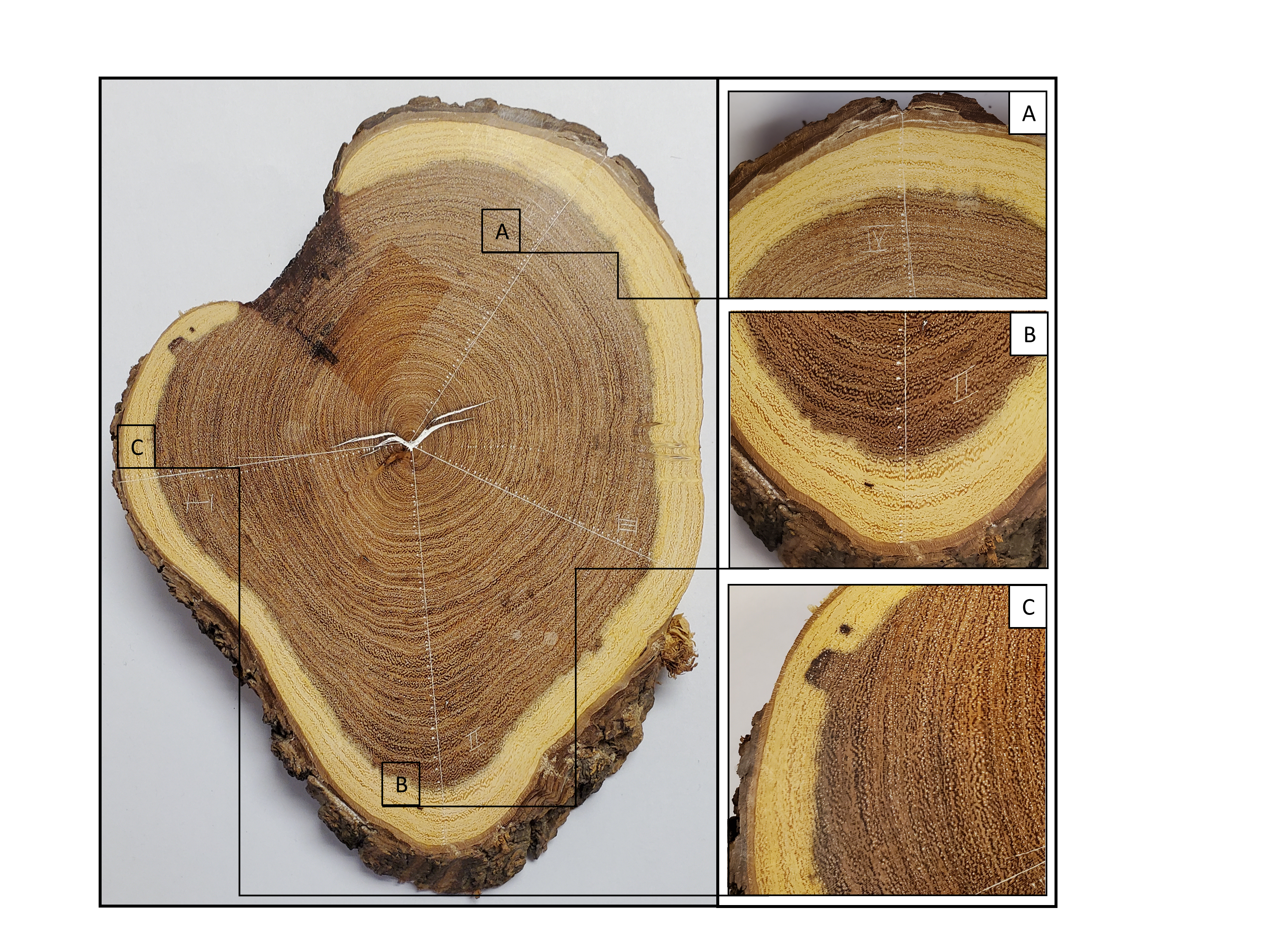

Supplement: Supplementary Figure 1 — Cross section of a sample of Neltuma alba, showing the eccentricity of the stem circumference. Zoomed area (A, B) show the variable length in terms of number of rings as the border between sapwood and heartwood doesn’t necessarily follow a single ring boundary. Zoomed area (C) show an extreme case of this same inconsistency of the border between wood types. [file Image_1.tif]
